# Suberoylanilide hydroxamic acid (SAHA) inhibits transforming growth factor-beta 2-induced increases in aqueous humor outflow resistance

**DOI:** 10.1016/j.jbc.2021.101070

**Published:** 2021-08-11

**Authors:** Tomokazu Fujimoto, Miyuki Inoue-Mochita, Satoshi Iraha, Hidenobu Tanihara, Toshihiro Inoue

**Affiliations:** 1 Department of Ophthalmology, Faculty of Life Sciences, Kumamoto University, Kumamoto, Japan; 2 Department of Medical Cell Biology, Institute of Molecular Embryology and Genetics, Kumamoto University, Kumamoto, Japan; 3 Kumamoto University Hospital, Kumamoto University, Kumamoto, Japan

**Keywords:** transforming growth factor beta (TGF-β), histone deacetylase inhibitor (HDAC inhibitor), eye, extracellular matrix protein, Akt, extracellular signal-regulated kinase (ERK), phosphatase and tensin homolog (PTEN), bone morphogenetic protein (BMP), α-SMA, alpha smooth muscle actin, BMP7, bone morphogenic protein 7, ECM, extra cellular matrix, ERK, extracellular signal-regulated kinase, HDAC, histone deacetylase, HTM, human trabecular meshwork, MSC, monkey Schlemm’s canal, MTM, monkey trabecular meshwork, PDPK1, 3-phosphoinositide-dependent protein kinase 1, PHLPP1, PH domain and leucine-rich repeat protein phosphatase 1, PI3K, phosphatidylinositol-3 kinase, PIK3CA, phosphatidylinositol-4,5-bisphosphate 3-kinase catalytic subunit alpha, PTEN, phosphatase and tensin homolog, RT-PCR, reverse transcription polymerase chain reaction, SAHA, suberoylanilide hydroxamic acid, SC, Schlemm’s canal, TDP-A, thailandepsin A, TEER, transendothelial electrical resistance, TGF-β2, transforming growth factor-beta 2, TM, trabecular meshwork, ZO-1, zonula occludens-1

## Abstract

Transforming growth factor-beta 2 (TGF-β2) is highly concentrated in the aqueous humor of primary open-angle glaucoma patients. TGF-β2 causes fibrosis of outflow tissues, such as the trabecular meshwork (TM), and increases intraocular pressure by increasing resistance to aqueous humor outflow. Recently, histone deacetylase (HDAC) activity was investigated in fibrosis in various tissues, revealing that HDAC inhibitors suppress tissue fibrosis. However, the effect of HDAC inhibitors on fibrosis in the eye was not determined. Here, we investigated the effect of suberoylanilide hydroxamic acid (SAHA), an HDAC inhibitor, on TGF-β2-induced increased resistance to aqueous humor outflow. We found that SAHA suppressed TGF-β2-induced outflow resistance in perfused porcine eyes. Moreover, SAHA cotreatment suppressed TGF-β2-induced ocular hypertension in rabbits. The permeability of monkey TM (MTM) and Schlemm’s canal (MSC) cell monolayers was decreased by TGF-β2 treatment. SAHA inhibited the effects of TGF-β2 on the permeability of these cells. TGF-β2 also increased the expression of extracellular matrix proteins (fibronectin and collagen type I or IV) in MTM, MSC, and human TM (HTM) cells, while SAHA inhibited TGF-β2-induced extracellular matrix protein expression in these cells. SAHA also inhibited TGF-β2-induced phosphorylation of Akt and ERK, but did not inhibit Smad2/3 phosphorylation, the canonical pathway of TGF-β signaling. Moreover, SAHA induced the expression of phosphatase and tensin homolog, a PI3K/Akt signaling factor, as well as bone morphogenetic protein 7, an endogenous antagonist of TGF-β. These results imply that SAHA prevents TGF-β2-induced increases in outflow resistance and regulates the non-Smad pathway of TGF-β signaling in TM and MSC cells.

Glaucoma, a major cause of blindness worldwide (1), is a multifactorial disorder (2, 3) in which elevated intraocular pressure (IOP) is a major risk factor (4, 5). The only evidence-based treatment for glaucoma is IOP reduction therapy *via* drugs or surgery. However, currently available treatments may not be sufficient to suppress visual field disorders; therefore new treatments are required. IOP is regulated by aqueous humor turnover in the anterior segment of the eye. IOP increases due to the inhibition of aqueous humor outflow through the trabecular meshwork (TM) and Schlemm’s canal (SC), possibly due to extracellular matrix (ECM) accumulation in the TM (6, 7). Recently, the relationship between epigenetic changes in the TM or SC and glaucoma has been investigated (8, 9, 10). Moreover, it was reported that steroid treatment, a risk factor for IOP elevation, changed the DNA methylation pattern in TM cells (11). Although these reports have suggested that epigenetic changes of the TM and SC are involved in regulating increased IOP, the mechanisms underlying the relationship between changes in IOP and epigenetics are unknown.

Histone deacetylase (HDAC) inhibitors were developed as anticancer drugs, and suberoylanilide hydroxamic acid (SAHA, vorinostat), romidepsin, and panobinostat are currently in clinical use (12). Recently, HDAC activity was investigated in fibrosis in various tissues, such as the lung, kidney, and liver, and it was shown that HDAC inhibitors suppressed tissue fibrosis (13, 14, 15, 16, 17, 18). In trabeculectomy (major glaucoma filtration surgery), postoperative maintenance of the newly created filtering bleb is important for IOP control. The filtering bleb works to regulate IOP by storing and absorbing aqueous humor, but its function is impaired when tissue scarring progresses over time following surgery. HDAC inhibitor prolonged bleb survival in a rabbit surgical model (19). Moreover, we reported that SAHA suppressed transforming growth factor-beta 2 (TGF-β2)-induced myofibroblast differentiation, cell proliferation, and ECM production *via* inhibition of Smad and non-Smad TGF-β signaling in human conjunctival fibroblast cells (20).

TGF-β2, which is highly concentrated in the aqueous humor of primary open-angle glaucoma patients (21, 22, 23, 24), induces an increase in aqueous outflow resistance (25, 26, 27). Moreover, TGF-β2 induces epithelial-to-mesenchymal transition (EMT)-like changes in TM cells, such as increased ECM production, actin stress fiber formation, and alpha smooth muscle actin (α-SMA) expression (27, 28, 29, 30, 31). We hypothesized that the HDAC inhibitor, which has an inhibitory effect on fibrosis in other tissues, would have the same effect on outflow tissue, such as TM and SC, and would increase the effect of SAHA on outflow resistance. We also examined the effect of SAHA on TGF-β2-induced changes in outflow tissue.

## Results

### The effect of SAHA on outflow facility and intraocular pressure

**Figure 1:**
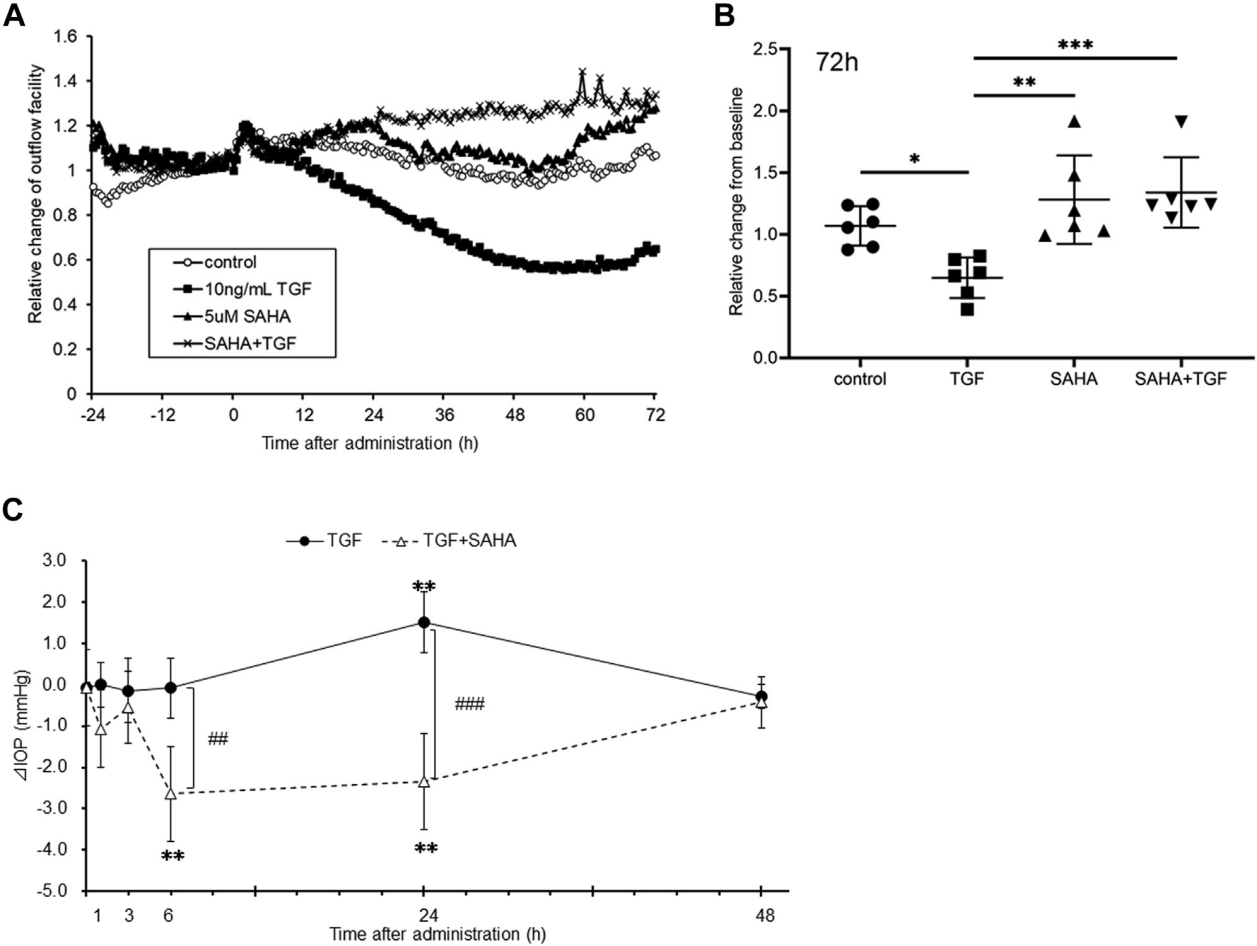
**Effect of SAHA on TGF-β2-induced outflow resistance and IOP elevation.***A* and *B*, the anterior segments of porcine eyes were perfused with 10 ng/ml TGF-β2 and/or 5 μM SAHA for 72 h. *A*, the time course of the outflow facility before and after drug perfusion is shown as the relative change from the baseline outflow facility. Data are presented as average values (n = 6). *B*, the graph shows the relative change in outflow facility compared with baseline after 72 h of drug perfusion. Data are presented as means ± SD (n = 6). ∗*p* < 0.05, ∗∗*p* < 0.01, and ∗∗∗*p* < 0.001, Tukey–Kramer HSD. *C*, IOPs of TGF-β2 or SAHA cotreatment in rabbit eyes were measured at each time point. The differences in IOP between the treated eye and the control eye (ΔIOP) are shown as mean ± standard deviation (SD, n = 5). ∗∗*p* < 0.01, Dunnett test *versus* pretreatment. ^##^*p* <0.01, ^###^*p* < 0.001, *t* test *versus* 2-group.

**Figure 2:**
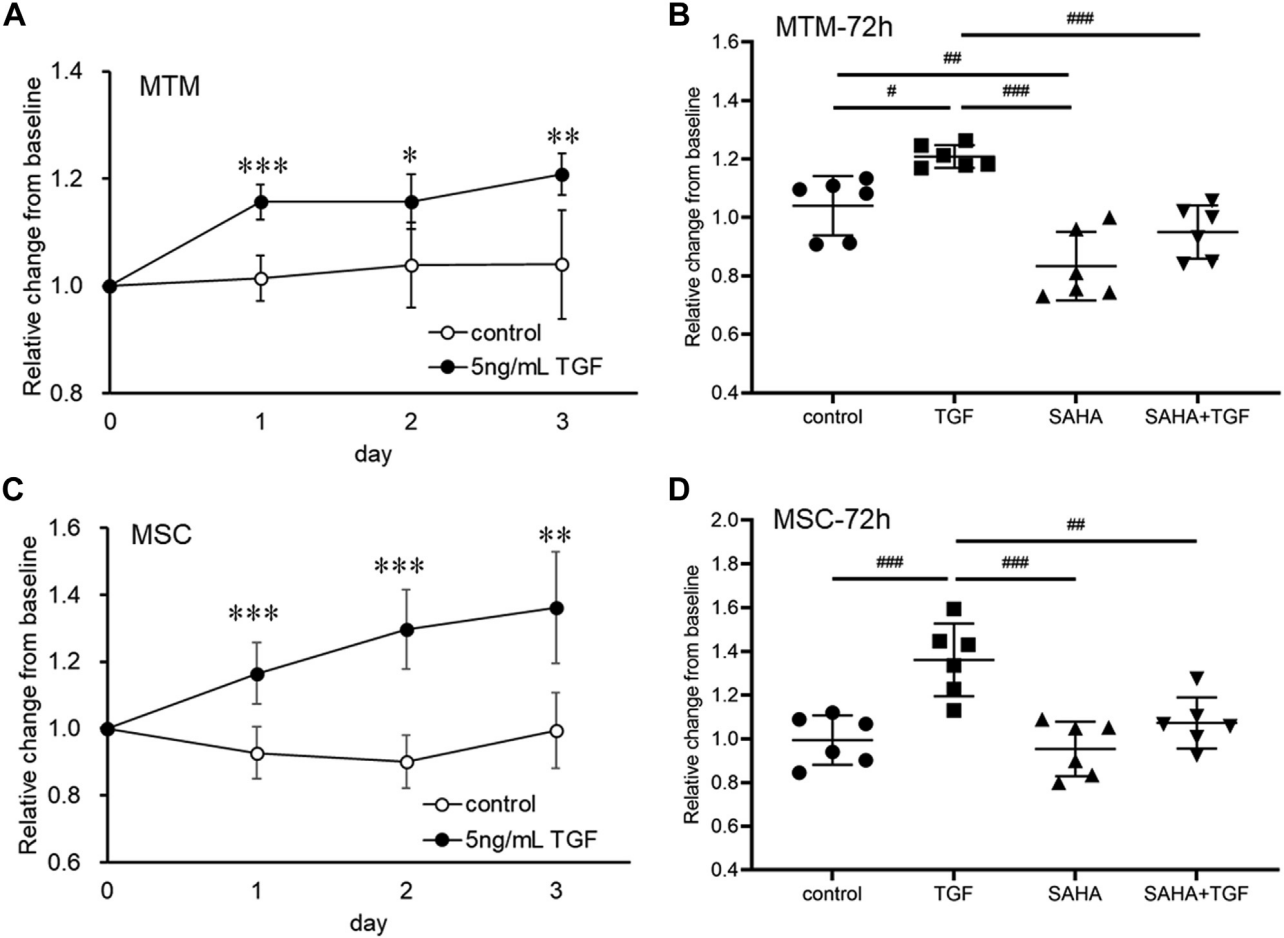
**The effects of TGF-β2 and SAHA on *trans*-endothelial electrical resistance (TEER) in monkey trabecular meshwork (MTM) and Schlemm’s canal endothelial (MSC) cells.** The time course of TEER after TGF-β2 treatment for 3 days on MTM (*A*) and MSC (*C*) is shown as the relative change from the baseline. Data are presented as means ± SD (n = 6). ∗*p* < 0.05, ∗∗*p* < 0.01, ∗∗∗*p* < 0.001, Student’s *t* test. MTM (*B*) and MSC (*D*) cells were treated with 5 ng/ml TGF-β2 and/or 5 μM SAHA for 72 h. The TEER values are shown as the relative change from the baseline. Data are presented as means ± SD (n = 6). ^#^*p* < 0.05, ^##^*p* < 0.01, ^###^*p* < 0.001, Tukey–Kramer HSD.

**Figure 3:**
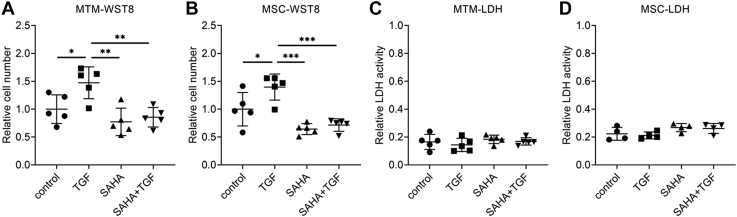
**Cell viability and cytotoxicity assays in MTM and MSC cells.** MTM and MSC cells were treated with 5 ng/ml TGF-β2 and/or 5 μM SAHA for 72 h. *A* and *B*, the cell viability of MTM (*A*) and MSC (*B*) cells was evaluated by WST-8 assay. *C* and *D*, the cytotoxicity of MTM (*C*) and MSC (*D*) was evaluated by lactate dehydrogenase (LDH) activity in the culture medium. Data are presented as means ± SD; n = 5 for the WST-8 assay; n = 4 for the LDH assay.

We evaluated the effects of SAHA, an HDAC inhibitor, on TGF-β2-induced outflow resistance using organ culture perfusion of a porcine eye. Perfusion of TGF-β2 decreased outflow facility over time (Fig. 1*A*). Perfusion of SAHA slightly increased outflow facility (outflow facility of the control at 72 h, 0.394 ± 0.062; SAHA, 0.578 ± 0.140 μl/min/mmHg). The simultaneous perfusion of TGF-β2 and SAHA significantly suppressed the TGF-β2-induced decrease in outflow facility at 72 h (Fig. 1*B*; outflow facility of TGF-β2 at 72 h, 0.272 ± 0.044; TGF-β2 + SAHA, 0.510 ± 0.117 μl/min/mmHg). Furthermore, we examined the effect of SAHA on TGF-β2-induced ocular hypertension in rabbits. The difference in IOP between the treated eye and contralateral eye (ΔIOP = treated eye IOP − contralateral eye IOP) was evaluated. The ΔIOPs before administration of TGF-β2 and TGF-β2 + SAHA were 0.06 ± 0.13 and −0.06 ± 0.93 mmHg, respectively (Fig. 1*C*, n = 5). Intracameral injection of TGF-β2 increased ΔIOP at 24 h compared with pretreatment (Fig. 1*C*, 1.52 ± 0.74 mmHg, *p* = 0.0013). By contrast, SAHA cotreatment reduced IOP at 6 h (−2.64 ± 1.14 mmHg, *p* = 0.0044) and 24 h (−2.34 ± 1.17 mmHg, *p* = 0.0013) compared with pretreatment. Next, we investigated the effects of SAHA on transendothelial electrical resistance (TEER) using monkey trabecular meshwork (MTM) and Schlemm’s canal endothelial (MSC) cells. TGF-β2 treatment increased TEER over time in MTM and MSC cells (Fig. 2, *A* and *C*). Simultaneous treatment with TGF-β2 and SAHA significantly suppressed TGF-β2-induced TEER elevation in MTM and MSC cells (Fig. 2, *B* and *D*). TGF-β2 resulted in significant proliferation of MTM and MSC cells, and simultaneous treatment with TGF-β2 and SAHA suppressed TGF-β2-induced cell proliferation (Fig. 3, *A* and *B*). TGF-β2 and SAHA showed no cytotoxicity under the same conditions as the TEER experiment in MTM and MSC cells (Fig. 3, *C* and *D*). These results indicate that SAHA acts directly on outflow tissue and suppresses the TGF-β2-induced elevation of outflow resistance.

### The effects of SAHA on the expression of ECM and cytoskeletal proteins

**Figure 4:**
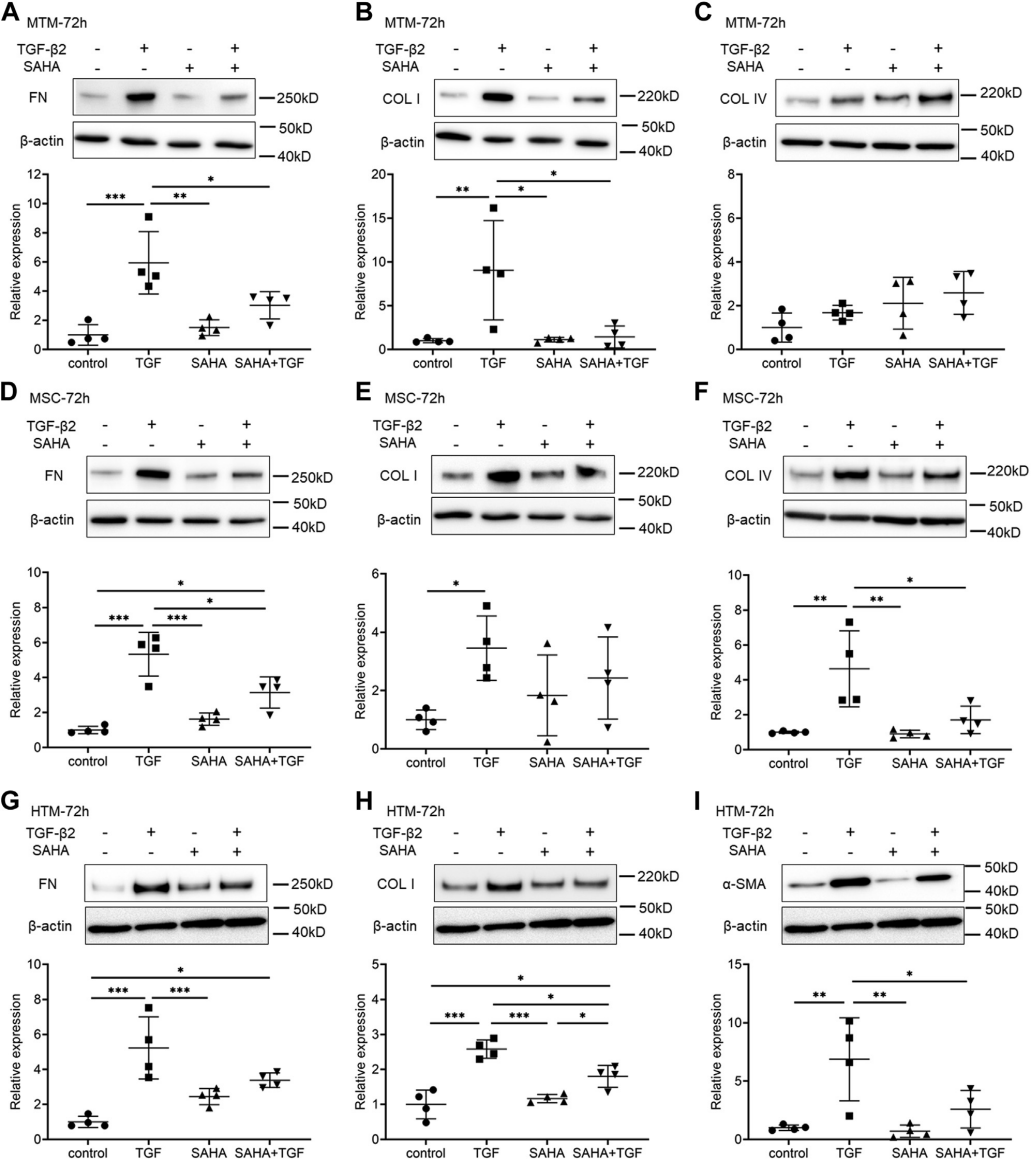
**The effects of TGF-β2 and SAHA on the expression of extracellular matrix protein in MTM, MSC, and HTM cells.** MTM (*A*–*C*), MSC (*D*–*F*), and HTM (*G*–*I*) cells were treated with 5 ng/ml TGF-β2 and/or 5 μM SAHA for 72 h. Fibronectin (FN; *A*, *D*, and *G*), collagen type I (COL I; *B*, *E*, and *H*), collagen type IV (COL IV; *C* and *F*), and α-SMA (*I*) expression levels were evaluated by western blotting. The WB bands displayed on *G*–*I* were obtained from the same membrane and display the same β-actin band as an endogenous control. Data are presented as means ± SD (n = 4). ∗*p* < 0.05, ∗∗*p* < 0.01, and ∗∗∗*p* < 0.001, Tukey–Kramer HSD.

**Figure 5:**
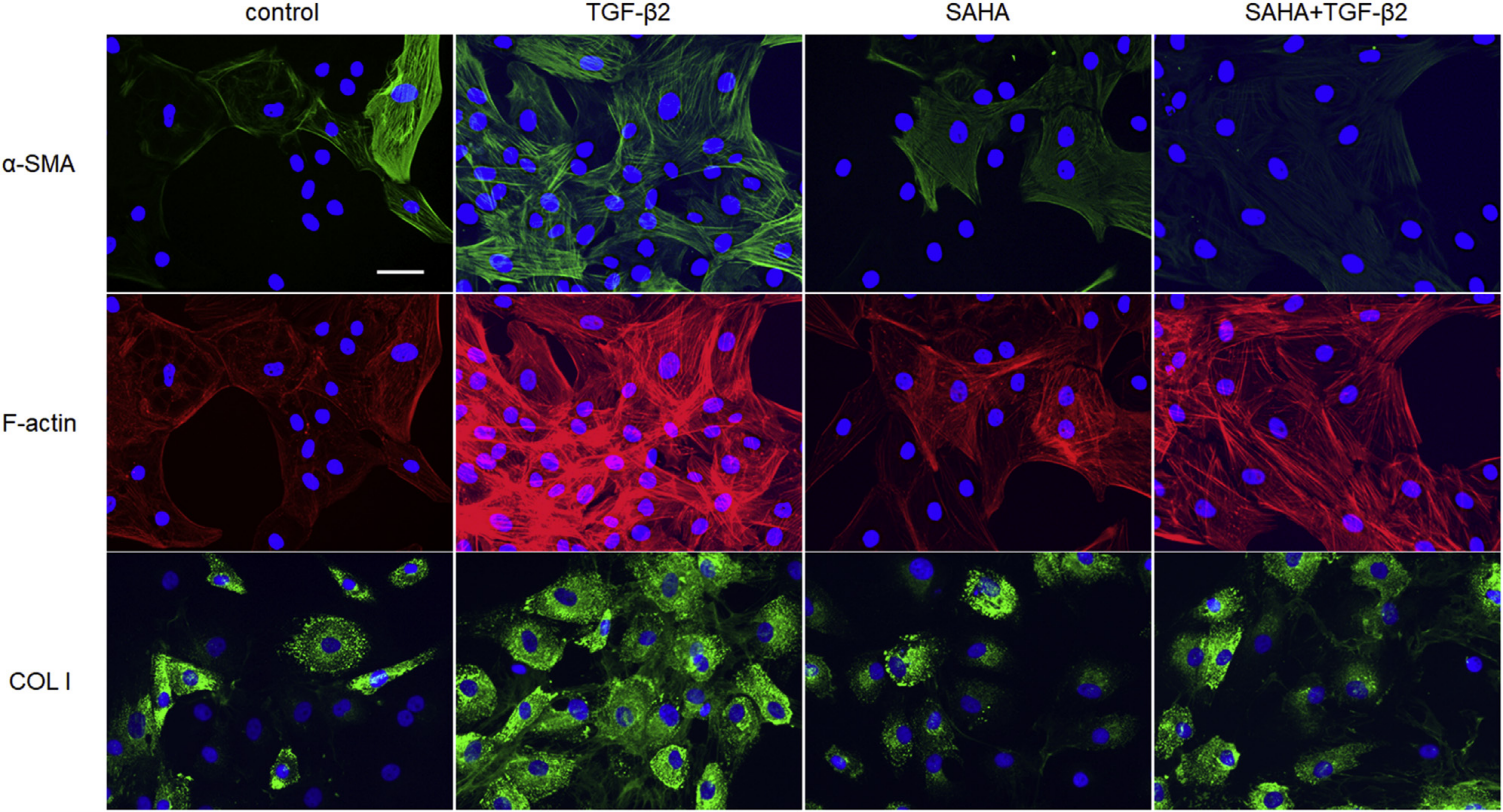
**Immunocytochemistry of MTM cells treated with TGF-β2 and SAHA.** The MTM cells were treated with 5 ng/ml TGF-β2 and 5 μM SAHA for 72 h. α-SMA (*top*, *green*), F-actin (*middle*, *red*), and collagen type I (COL I; *bottom*, *green*) were visualized by immunostaining. Cell nuclei were counterstained with DAPI (*blue*). Scale bar = 50 μm.

**Figure 6:**
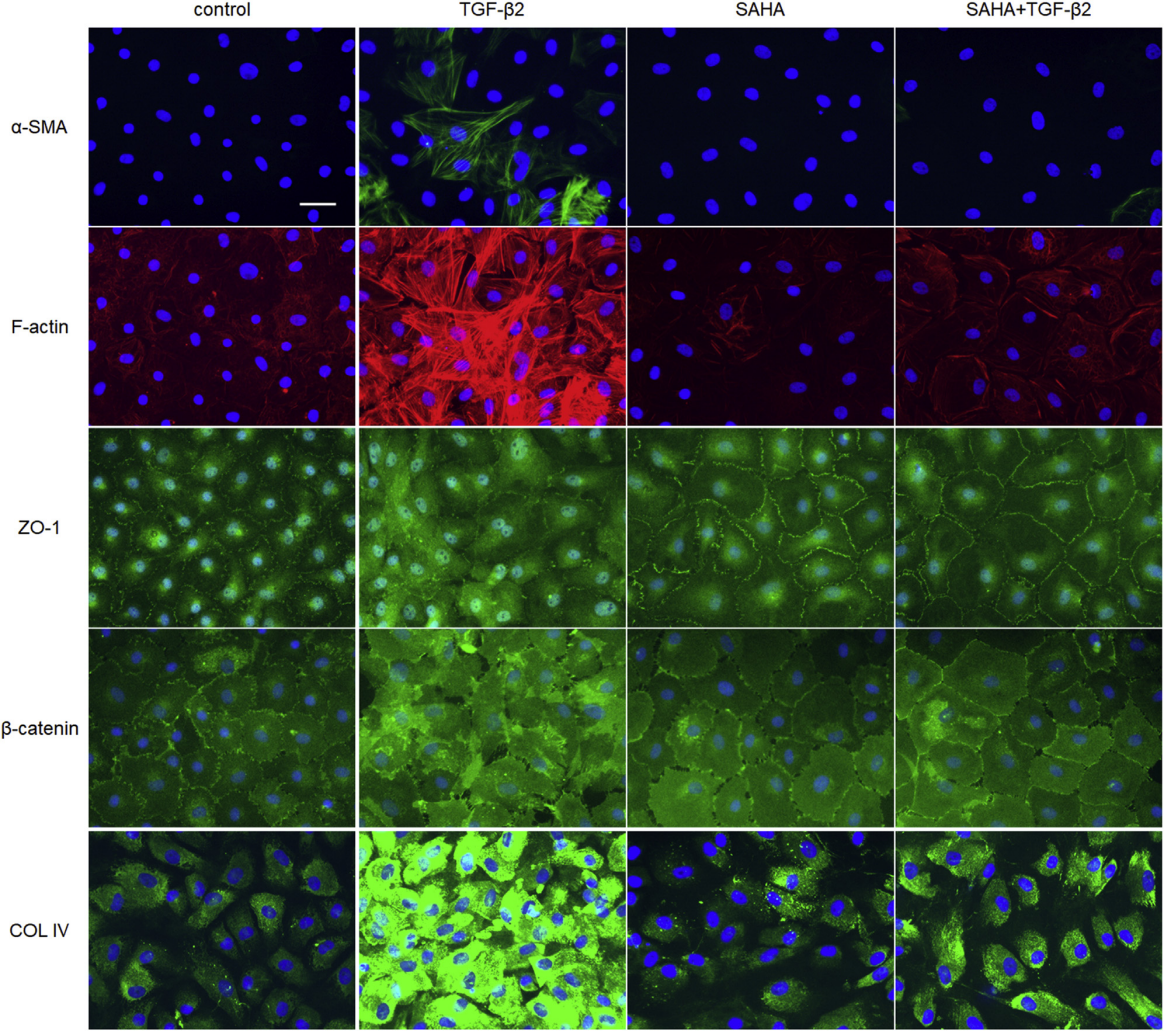
**Immunohistochemistry of MSC cells treated with TGF-β2 and SAHA.** The MSC cells were treated with 5 ng/ml TGF-β2 and 5 μM SAHA for 72 h. α-SMA (*top*, *green*), F-actin (*second*, *red*), ZO-1 (*third*, *green*), β-catenin (*fourth*, *green*), and collagen type IV (COL IV; *bottom*, *green*) were visualized by immunostaining. Scale bar = 50 μm.

**Figure 7:**
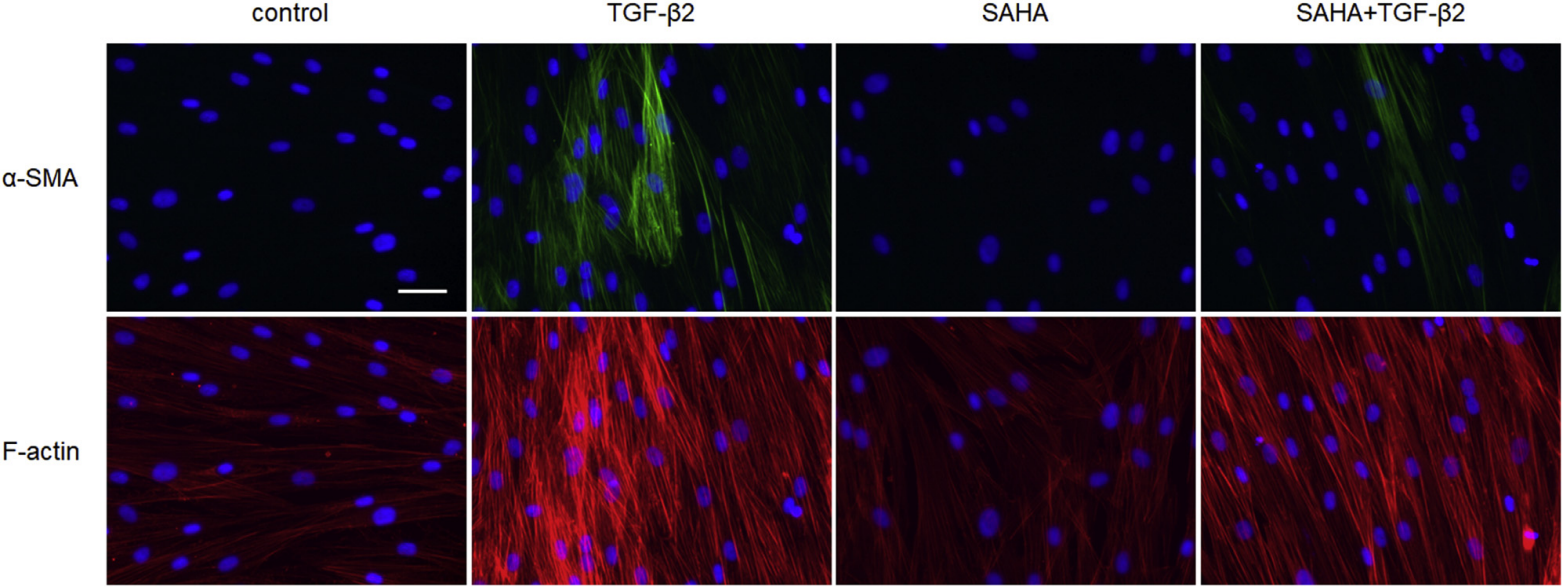
**Immunocytochemistry of HTM cells treated with TGF-β2 and SAHA.** The HTM cells were treated with 5 ng/ml TGF-β2 and 5 μM SAHA for 72 h. α-SMA (*top*, *green*) and F-actin (*bottom*, *red*) were visualized by immunostaining. Cell nuclei were counterstained with DAPI (*blue*). Scale bar = 50 μm.

We evaluated the effects of SAHA on ECM and cytoskeletal proteins, which affect outflow resistance, in MTM, MSC, and human trabecular meshwork (HTM) cells. HDAC inhibition of SAHA was confirmed by western blotting on the acetylation of histones H3 and H4 in MTM and MSC cells (Fig. S1). The protein expression and mRNA expression of fibronectin and collagen types I and IV were evaluated by western blotting and real-time reverse transcription PCR (RT-PCR). In MTM cells, TGF-β2 increased the expression of fibronectin and collagen type I, and SAHA suppressed this TGF-β2-induced elevation (Fig. 4, *A*–*C*). In MSC cells, TGF-β2 increased the expression of fibronectin and collagen type IV, and SAHA suppressed this TGF-β2-induced elevation (Fig. 4, *D*–*F*). In HTM cells, SAHA suppressed the TGF-β2-induced elevation of fibronectin and collagen type I expression (Fig. 4, *G* and *H*). Expression of α-SMA protein was significantly increased by TGF-β2 treatment, and SAHA suppressed the TGF-β2-induced α-SMA expression in HTM cells (Fig. 4*I*). Moreover, the results of ECM and α-SMA mRNA expression were similar to those of western blotting (Fig. S2). The immunocytochemistry analyses showed that SAHA suppressed α-SMA and actin stress fibers whose expression was increased by TGF-β2 in MTM, MSC, and HTM cells (Figure 5, Figure 6, Figure 7). The fluctuations in expression of collagen types I and IV due to TGF-β2 and SAHA were also confirmed by immunocytochemistry in MTM and MSC cells (Figs. 5 and 6). Zonula occludens-1 (ZO-1) and β-catenin, which are cell–cell adhesion factors, displayed obvious changes in their staining properties following TGF-β2 stimulation (Fig. 6). TGF-β2 stimulation increased the stainability of the entire cell, while simultaneous treatment with TGF-β2 and SAHA suppressed the TGF-β2-induced increase in stainability.

### The effects of SAHA on EMT or endothelial-to-mesenchymal transition

**Figure 8:**
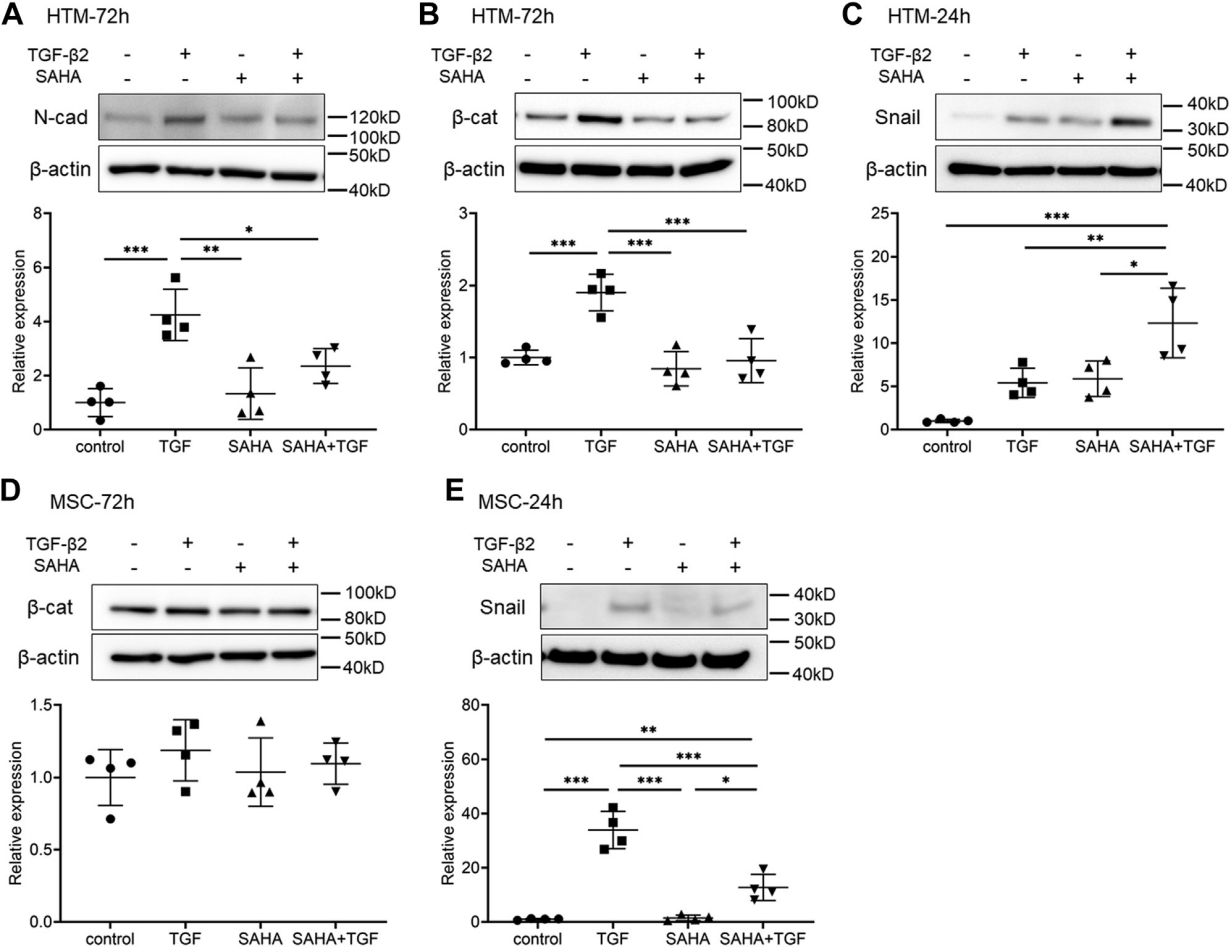
**The effects of SAHA on TGF-β2-induced EMT and End-MT-like changes in HTM and MSC cells.** HTM (*A*–*C*) and MSC (*D* and *E*) cells were treated with 5 ng/ml TGF-β2 and/or 5 μM SAHA for 24 and 72 h. N-cadherin (*A*), β-catenin (*B* and *D*), and Snail (*C* and *E*) expression levels were evaluated by western blotting. Data are presented as means ± SD (n = 4). ∗*p* < 0.05, ∗∗*p* < 0.01, and ∗∗∗*p* < 0.001, Tukey–Kramer HSD.

Studies have so far shown that SAHA suppresses EMT-like changes such as TGF-β2-induced upregulation of α-SMA and fibronectin as well as cell proliferation in TM cells. Similarly, SAHA suppresses endothelial-to-mesenchymal transition (End-MT)-like changes including TGF-β2-induced upregulation of fibronectin and α-SMA in MSC cells. Therefore, we conducted further studies on EMT and End-MT-like phenomena in HTM and MSC cells (Fig. 8). SAHA significantly suppressed TGF-β2-induced upregulation of N-cadherin, a mesenchymal marker, and β-catenin in HTM cells (Fig. 8, *A* and *B*). However, the expression of Snail, an EMT inducer, was not suppressed by SAHA treatment, and simultaneous treatment with SAHA and TGF-β2 caused a significant increase in Snail expression (Fig. 8*C*). On the other hand, the expression level of β-catenin did not change with TGF-β2 or SAHA treatment in MSC cells (Fig. 8*D*). In addition, TGF-β2 increased the expression of Snail, and SAHA significantly suppressed TGF-β2-induced elevation of Snail expression in MSC cells (Fig. 8*E*).

### The effects of SAHA on Smad and non-Smad TGF-β signaling

**Figure 9:**
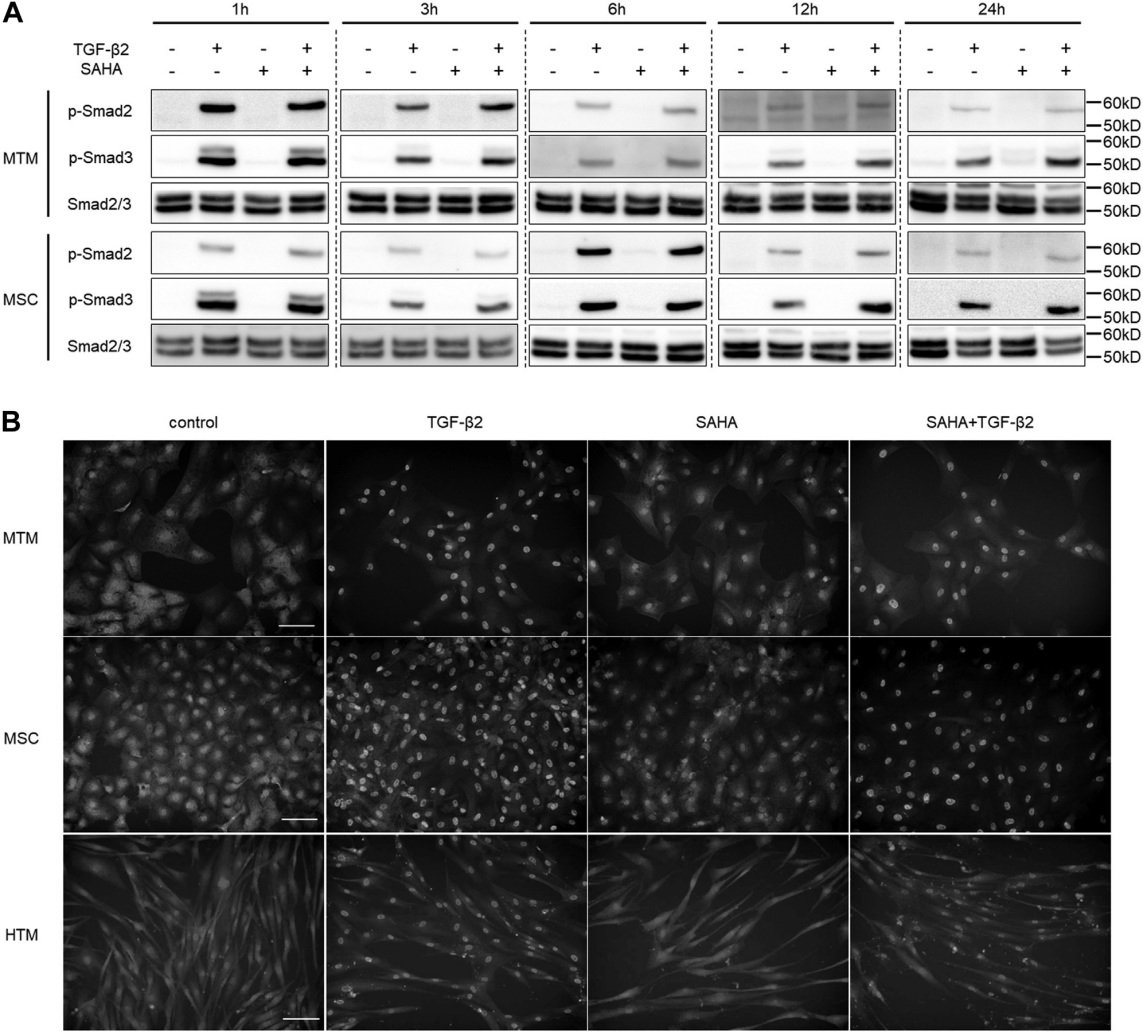
**The effects of SAHA on TGF-β2-induced Smad phosphorylation in MTM, MSC, and HTM cells.***A*, MTM and MSC cells were treated with 5 ng/ml TGF-β2 and 5 μM SAHA for 1, 3, 6, 12, and 24 h. The representative bands of phospho-Smad2 (p-Smad2), phospho-Smad3 (p-Smad3), and total Smad2/3 are shown. *B*, MTM, MSC, and HTM cells were treated with 5 ng/ml TGF-β2 and 5 μM SAHA for 72 h. Nuclear localization of Smad2/3 was evaluated by immunostaining. Scale bar = 100 μm.

**Figure 10:**
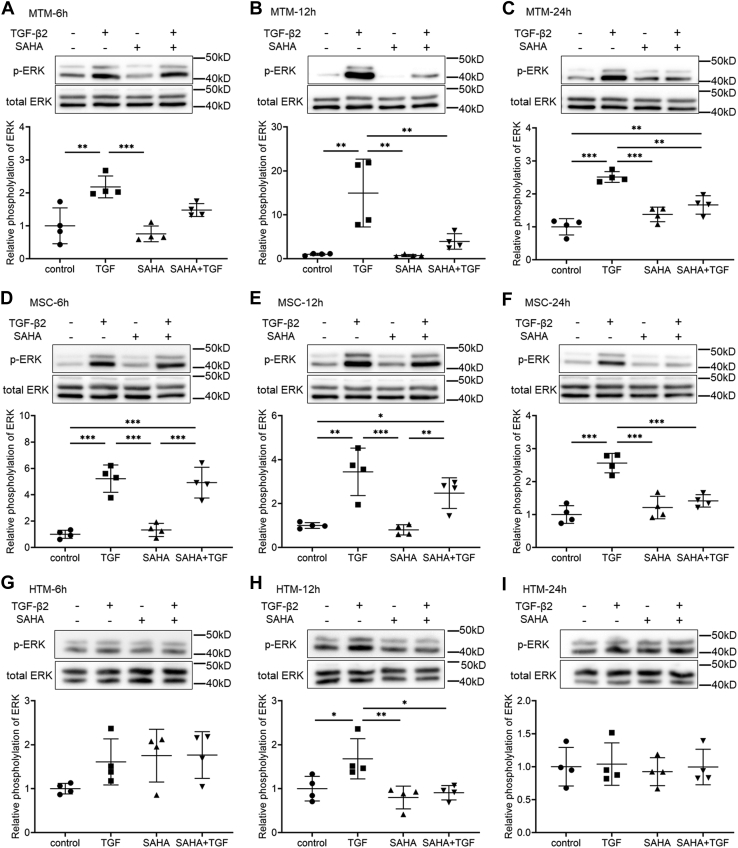
**The effects of SAHA on TGF-β2-induced ERK phosphorylation in MTM, MSC, and HTM cells.** MTM (*A*–*C*), MSC (*D*–*F*), and HTM (*G*–*I*) cells were treated with TGF-β2 and SAHA for 6 h (*A*, *D*, and *G*), 12 h (*B*, *E*, and *H*), and 24 h (*C*, *F*, and *I*). The *top* of each figure indicates the representative band of phospho-ERK (p-ERK). Data for the relative phosphorylation are presented as means ± SD (n = 4). ∗*p* < 0.05, ∗∗*p* < 0.01, and ∗∗∗*p* < 0.001, Tukey–Kramer HSD.

**Figure 11:**
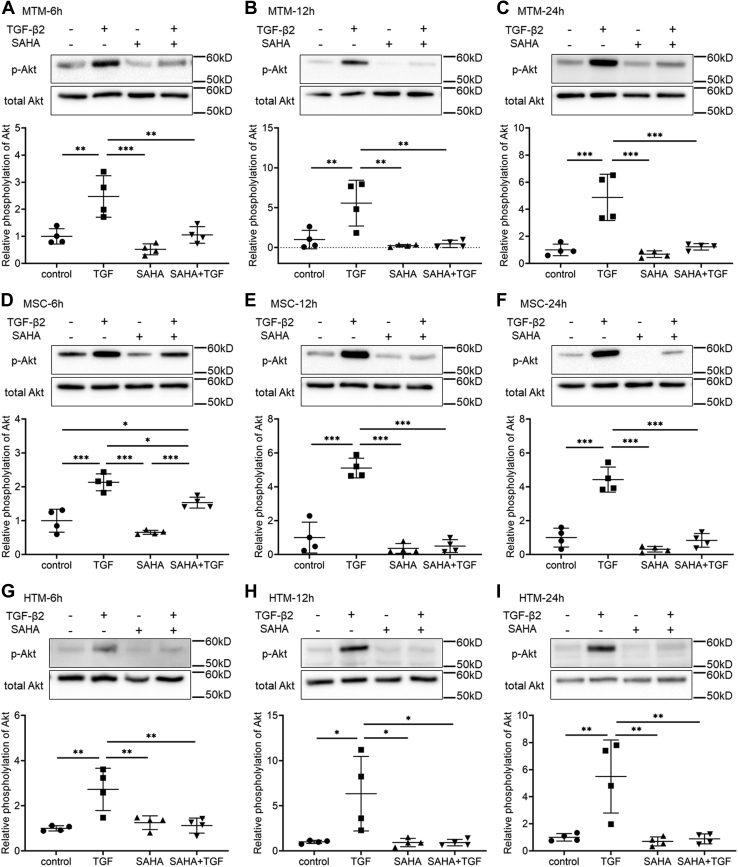
**The effects of SAHA on TGF-β2-induced Akt phosphorylation in MTM, MSC, and HTM cells.** MTM (*A*–*C*), MSC (*D*–*F*), and HTM (*G*–*I*) cells were treated with TGF-β2 and SAHA for 6 h (*A*, *D*, and *G*), 12 h (*B*, *E*, and *H*), and 24 h (*C*, *F*, and *I*). The *top* of each figure indicates the representative band of phospho-Akt (p-Akt). Data for the relative phosphorylation are presented as means ± SD (n = 4). ∗*p* < 0.05, ∗∗*p* < 0.01, and ∗∗∗*p* < 0.001, Tukey–Kramer HSD.

We evaluated the effects of SAHA on the TGF-β2 signaling pathway. SAHA did not affect the TGF-β2-induced phosphorylation of Smad in MTM or MSC cells at any of the times tested (Fig. 9*A*). Moreover, SAHA did not affect the TGF-β2-induced nuclear localization of Smad in MTM, MSC, or HTM cells (Fig. 9*B*). Subsequently, the effects of SAHA on non-Smad signaling were evaluated at 6, 12, and 24 h after TGF-β2 and SAHA treatment. In MTM cells, TGF-β2 induced extracellular signal-regulated kinase (ERK) phosphorylation at all time points, and SAHA significantly reduced TGF-β2-induced ERK phosphorylation at 12 and 24 h (Fig. 10, *A*–*C*). In MSC cells, ERK phosphorylation by TGF-β2 was the same as in MTM cells and SAHA significantly reduced ERK phosphorylation by TGF-β2 at 24 h (Fig. 10, *D*–*F*). In HTM cells, SAHA significantly reduced ERK phosphorylation induced by TGF-β2 treatment (Fig. 10, *G*–*I*). The effects of SAHA on Akt phosphorylation were more pronounced than on ERK phosphorylation; SAHA significantly reduced TGF-β2-induced Akt phosphorylation at 6, 12, and 24 h after treatment in MTM, HTM, and MSC cells (Fig. 11).

### Involvement of the phosphatidylinositol-3 kinase (PI3K)/Akt pathway in response to SAHA

**Figure 12:**
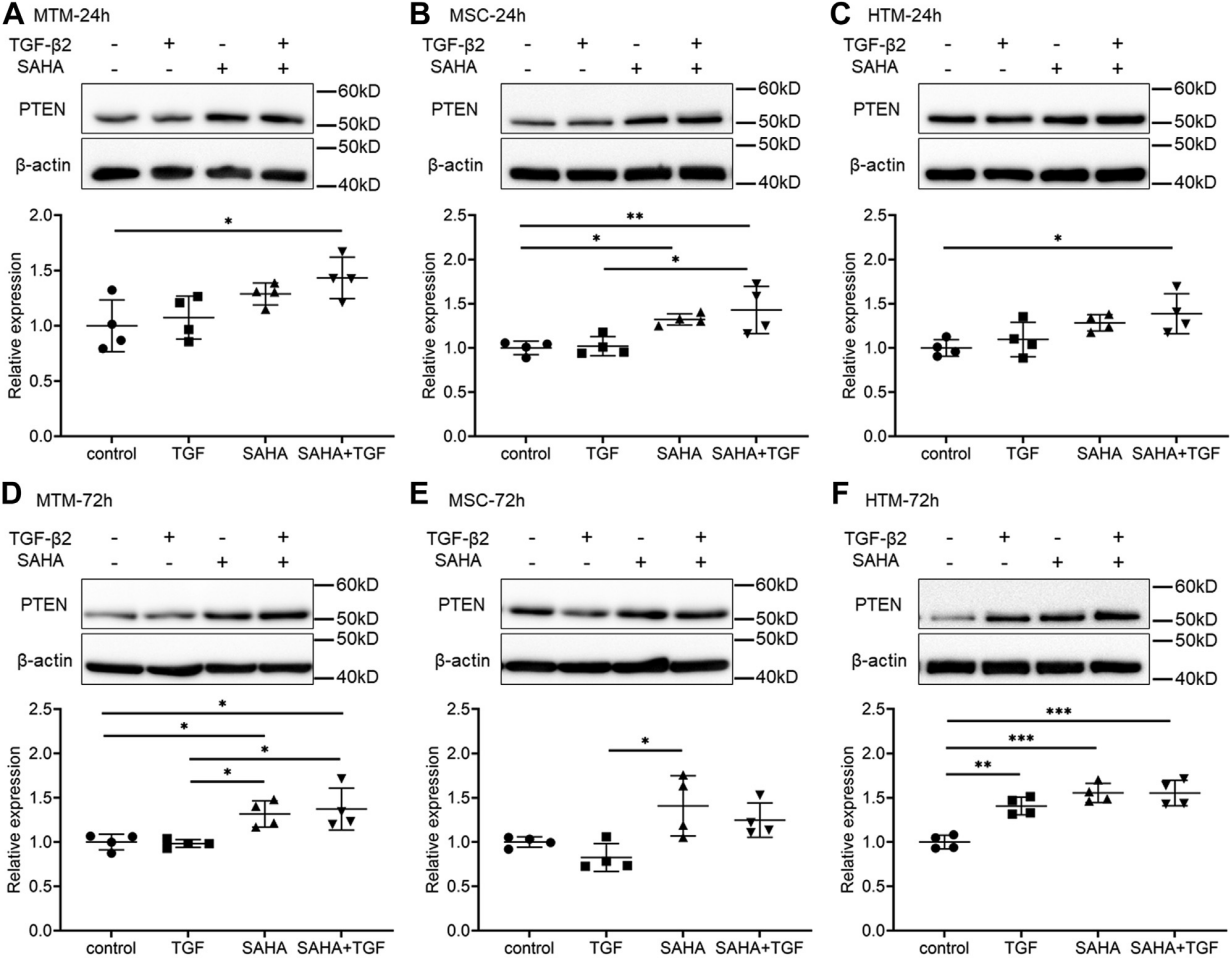
**The effects of TGF-β2 and SAHA on PTEN expression in MTM, MSC, and HTM cells.** The cells were treated with 5 ng/ml TGF-β2 and 5 μM SAHA for 24 h (*A*–*C*) or 72 h (*D*–*F*). The expression levels of PTEN in MTM (*A* and *D*), MSC (*B* and *E*), and HTM (*C* and *F*) cells after TGF-β2 and SAHA treatment were evaluated by western blotting. Data are presented as means ± SD (n = 4). ∗*p* < 0.05, ∗∗*p* < 0.01, and ∗∗∗*p* < 0.001, Tukey–Kramer HSD.

**Figure 13:**
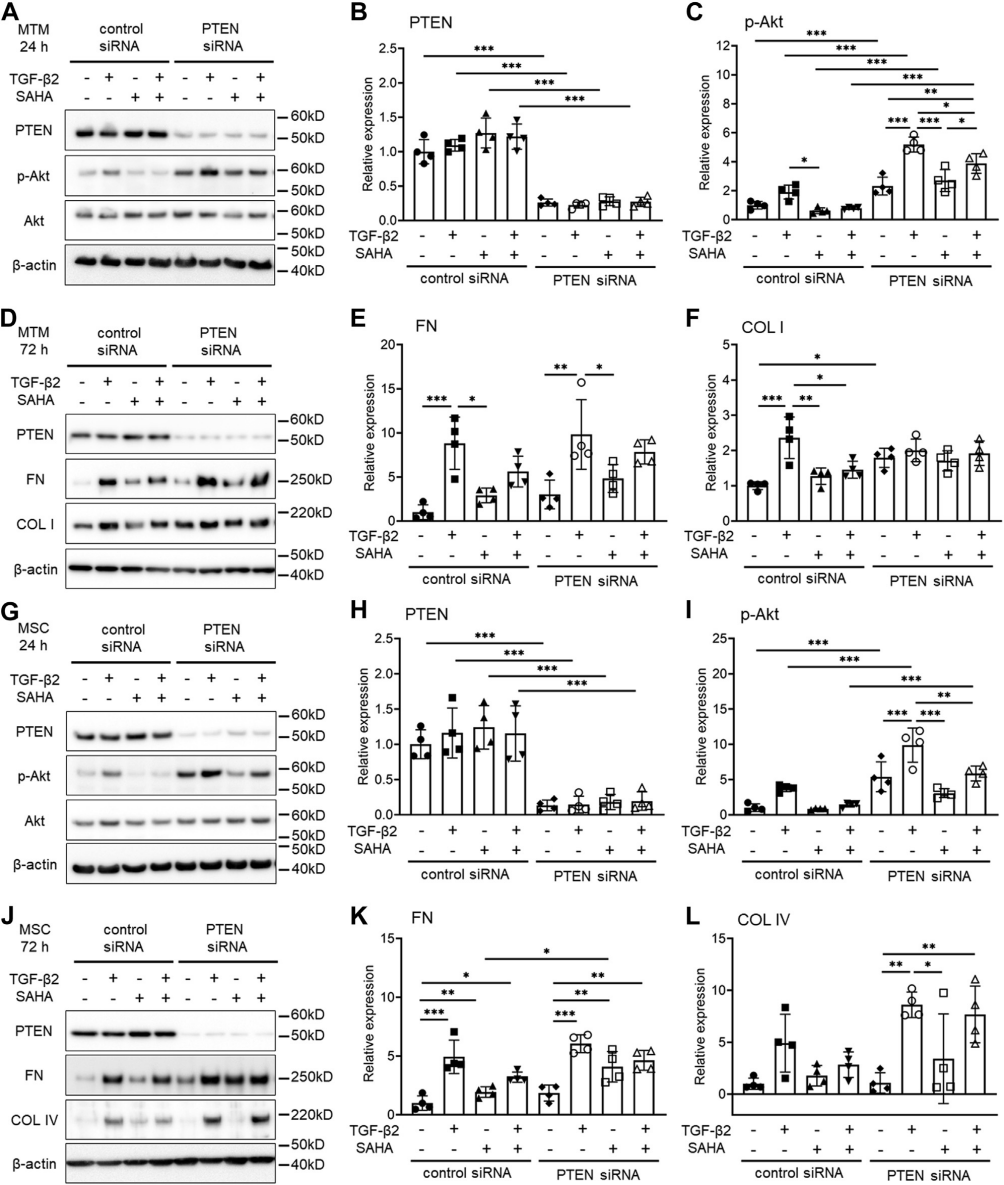
**The effects of PTEN knockdown on protein expression after TGF-β2 and SAHA treatment of MTM and MSC cells.** Control and PTEN siRNA-transfected MTM and MSC cells were treated with 5 ng/ml TGF-β2 and 5 μM SAHA for 24 h and 72 h. *A*, the representative PTEN, p-Akt, and Akt bands of the MTM cells after TGF-β2 and SAHA treatment for 24 h. *B* and *C*, relative expression of PTEN (*B*) and p-Akt (*C*) in MTM cells after TGF-β2 and SAHA treatment for 24 h. *D*, the representative PTEN, fibronectin (FN), and collagen type I (COL I) bands of MTM cells after TGF-β2 and SAHA treatment for 72 h. *E* and *F*, relative expression of FN (*E*) and COL I (*F*) in MTM cells after treatment for 72 h. *G*, the representative PTEN, p-Akt, and Akt bands of MSC cells after TGF-β2 and SAHA treatment for 24 h. *H* and *I*, relative expression of PTEN (*H*) and p-Akt (*I*) in MSC cells after TGF-β2 and SAHA treatment for 24 h. *J*, the representative PTEN, fibronectin (FN), and collagen type I (COL I) bands of MSC cells after TGF-β2 and SAHA treatment for 72 h. *K* and *L*, relative expression of FN (*K*) and COL I (*L*) in MSC cells after treatment for 72 h. Data are presented as means ± SD (n = 4). ∗*p* < 0.05, ∗∗*p* < 0.01, and ∗∗∗*p* < 0.001, Tukey–Kramer HSD.

Since SAHA had a significant inhibitory effect on Akt phosphorylation, we investigated changes in the expression of phosphatase and tensin homolog (PTEN), which acts as an upstream regulator of the PI3K/Akt signaling pathway. In MTM and HTM cells, simultaneous treatment with TGF-β2 and SAHA significantly increased PTEN expression at 24 h after treatment (Fig. 12, *A* and *C*). At 72 h after treatment, both SAHA alone and simultaneous treatment with TGF-β2 significantly increased expression of PTEN (Fig. 12, *D* and *F*). In MSC cells, both SAHA alone and simultaneous treatment with TGF-β2 significantly increased PTEN expression at 24 h after treatment, and these effects were sustained for 72 h (Fig. 12, *B* and *E*). Next, we used PTEN knockdown MTM and MSC cells to investigate the role of PTEN on the effects of SAHA. In MTM cells, PTEN knockdown using small interfering RNA (siRNA) transfection significantly increased Akt phosphorylation compared with control siRNA transfection (Fig. 13, *A*–*C*). Fibronectin expression was increased by PTEN knockdown, and SAHA did not affect TGF-β2-induced fibronectin expression in PTEN knockdown MTM cells (Fig. 13, *D* and *E*). Collagen type I expression was significantly increased by PTEN knockdown in MTM cells and SAHA did not affect collagen type I expression in PTEN knockdown MTM cells (Fig. 13, *D* and *F*). In MSC cells, PTEN knockdown using siRNA transfection also significantly increased Akt phosphorylation compared with control siRNA transfection (Fig. 13, *G*–*I*). The inhibitory effect of SAHA on TGF-β2-induced fibronectin expression was decreased by PTEN knockdown in MSC cells, and fibronectin expression levels following SAHA treatment were significantly higher in PTEN knockdown MSC cells than in the control (Fig. 13, *J* and *K*). PTEN knockdown increased the TGF-β2-induced elevation of collagen type IV expression in MSC cells and SAHA did not affect the TGF-β2-induced elevation of collagen type IV expression (Fig. 13, *J* and *L*). The expression of α-SMA (*ACTA2*) mRNA was increased by PTEN knockdown in MTM cells (Fig. S3*A*). In MSC cells, PTEN knockdown increased the basal level of *ACTA2* expression and the TGF-β2-induced elevation of *ACTA2* expression. However, SAHA tended to suppress the TGF-β2-induced *ACTA2* expression in PTEN knockdown MSC cells (Fig. S3*E*). PTEN knockdown did not affect the mRNA expression of 3-phosphoinositide dependent protein kinase 1 (*PDPK1*), PH domain and leucine-rich repeat protein phosphatase 1 (*PHLPP1*), or phosphatidylinositol 3-kinase catalytic subunit alpha (*PIK3CA*), involved in PI3K/Akt signaling (Fig. S3).

### Induction of bone morphogenetic protein 7 (BMP7) by SAHA

**Figure 14:**
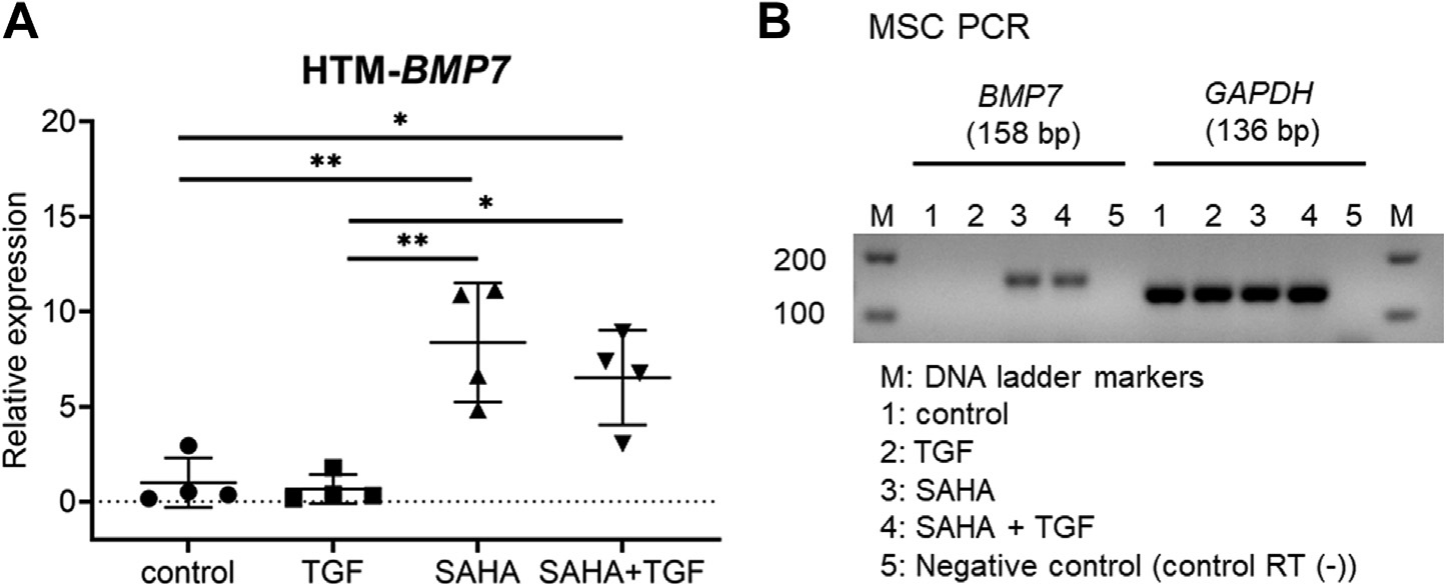
**SAHA induced *BMP7* mRNA expression in HTM and MSC cells.***A*, HTM cells were treated with 5 ng/ml TGF-β2 and 5 μM SAHA for 24 h. The mRNA expression levels of *BMP7* were evaluated by real-time PCR. Data are presented as means ± SD (n = 4). ∗*p* < 0.05, ∗∗*p* < 0.01, and ∗∗∗*p* < 0.001, Tukey–Kramer HSD. *B*, MSC cells were treated with 5 ng/ml TGF-β2 and 5 μM SAHA for 24 h. The mRNA expression levels of *BMP7* were evaluated by real-time PCR. Since no expression of *BMP7* was observed under the control conditions, the electrophoresis results of the PCR product are shown. Similar results were acquired from four independent samples. Control RT (−), control sample without reverse transcription.

HDAC inhibitors induce BMP7 and inhibit TGF-β-induced renal fibrosis (32, 33). In addition, BMP7 inhibits TGF-β2-induced ECM expression in HTM cells (34). Therefore, we examined *BMP7* mRNA expression in HTM and MSC cells *via* real-time RT-PCR. In HTM cells, *BMP7* was expressed under normal conditions, and its expression was significantly increased following SAHA treatment (Fig. 14*A*). Increased *BMP7* expression was also observed following cotreatment with TGF-β2 and SAHA. On the other hand, in MSC cells, *BMP7* expression was not detected under control or TGF-β2 treatment conditions; however, SAHA alone or cotreatment with SAHA and TGF-β2 induced *BMP7* mRNA expression (Fig. 14*B*).

## Discussion

Our results indicate that SAHA acts directly on TM and SC cells and suppresses TGF-β2-induced elevation of outflow resistance. SAHA suppressed the TGF-β2-induced elevation of ECM and cytoskeletal protein expression in TM and SC cells, and we think that the mechanisms underlying this suppression include inhibition of Akt and ERK phosphorylation by TGF-β2. ERK activation induces increased actin stress fibers, α-SMA, and fibronectin expression in TM cells (30). ERK inhibition also suppresses TGF-β-induced expression of α-SMA, collagen, and fibronectin in conjunctival, lung, and cardiac fibroblast cells (35, 36, 37). However, other reports showed that inhibition of ERK activity did not affect the expression of actin stress fibers or α-SMA in TM cells (31, 38), but induced fibrosis similar to the increased expression of α-SMA and collagen in dermal fibroblasts (39). Since different effects of ERK inhibition have been reported, further studies are needed on the effects of SAHA-induced ERK inhibition.

Activation of the PI3K/Akt pathway is related to the expression of α-SMA in TM cells (31). Moreover, the PI3K/Akt pathway is involved in fibronectin and collagen expression in fibroblasts (35, 40, 41, 42). We focused on PI3K/Akt signaling, which was significantly affected by SAHA, and confirmed that SAHA increased the expression of PTEN. PTEN dephosphorylates phosphatidylinositol 3,4,5-trisphosphate and suppresses the PI3K/Akt pathway. PTEN knockdown inhibited the suppressive effect of SAHA on the TGF-β2-induced elevated expression of collagen types I and IV in MTM and MSC cells, respectively. However, the inhibitory effect of SAHA on Akt phosphorylation remained under PTEN knockdown conditions. These data suggest that the mechanism of the inhibitory effect of SAHA on Akt phosphorylation could not be explained by the induction of PTEN expression alone, and further studies are needed for clarification.

ZO-1 and β-catenin are cell–cell adhesion factors that showed increased staining in whole MSC cells following TGF-β2 stimulation (Fig. 6). By contrast, treatment of SAHA with TGF-β2 reduced the expression of both proteins throughout the cell, and expression near the cell membrane was observed. In previous reports, ERK or Akt inhibitors reduced the TGF-β induced cell–cell adhesion *via* β-catenin in TM cells (43). Our results for ZO-1 and β-catenin may also be due to ERK and Akt signaling inhibition by SAHA.

In addition, β-catenin acts as a signal transduction molecule for the Wnt/β-catenin signal, which induces EMT (44). In this study, TGF-β2 increased the expression level of β-catenin in TM cells. The expression of N-cadherin, an EMT marker, was also elevated, suggesting that TGF-β2 induced an EMT-like phenomenon in TM cells. In addition, SAHA reduced expression of TGF-β2-induced EMT marker proteins such as fibronectin, N-cadherin, and α-SMA in TM cells. However, the expression of Snail, an EMT inducer, was increased by SAHA treatment, suggesting that SAHA may not fully suppress EMT. In MSC cells, SAHA significantly suppressed the induction of Snail expression by TGF-β2. Given the fluctuations in the intracellular distributions of ZO-1 and β-catenin, as well as in the expression levels of fibronectin and α-SMA, SAHA most likely suppressed TGF-β2-induced End-MT in MSC cells.

BMP7 is a member of the TGF-β superfamily of cytokines and acts as an endogenous antagonist of TGF-β (44, 45). BMP7 also inhibits the action of TGF-β in several cell types including TM cells (34, 46, 47, 48, 49). In addition, HDAC inhibitors induce BMP7 and inhibit TGF-β-induced renal fibrosis (32, 33). In this study, we found that the HDAC inhibitor SAHA induced *BMP7* mRNA expression in TM and SC cells. Considering previous reports and the findings from this study, the induction of BMP7 expression by SAHA is probably strongly involved in the inhibitory effect of SAHA on the action of TGF-β2 in TM and SC cells.

Thailandepsin A (TDP-A), an HDAC inhibitor, induces TGF-β2 expression in TM cells and increases IOP (50). By contrast, our results show that SAHA increased outflow facility and suppressed TGF-β2-induced reduction of outflow facility in organ culture perfusion tests. SAHA also suppresses changes caused by TGF-β in cells other than TM (20, 51, 52, 53, 54). However, the effects of TDP-A have been confirmed only in cancer cells, and there are no reports of its effects on fibroblasts (55, 56, 57). The reasons for the different reactions to drugs within the same category are unknown, but our results imply that SAHA acts by suppressing TGF-β-induced changes.

In conclusion, SAHA prevents TGF-β2-induced increases in outflow resistance and regulates the non-Smad pathway of TGF-β signaling in MTM, MSC, and HTM cells. These results imply that HDAC inhibition may be a potential new target for IOP-reducing agents.

## Experimental procedures

### Anterior segment organ culture perfusion

The perfusion protocol was described previously (58, 59). Enucleated porcine eyes were purchased from a local abattoir and cut at the equator, and the vitreous, lens, iris, and ciliary body were removed from the anterior segment. The anterior segments were placed in custom-designed chambers and perfused with high-glucose Dulbecco’s modified Eagle medium (DMEM; Sigma-Aldrich, Merck KGaA) in the presence of 0.1% fetal bovine serum (FBS; Hyclone, Thermo Fisher Scientific), penicillin (100 U/ml; Thermo Fisher Scientific), streptomycin (100 μg/ml; Thermo Fisher Scientific), amphotericin B (0.25 μg/ml; Thermo Fisher Scientific), and gentamicin (36 μg/ml; Thermo Fisher Scientific) at a constant flow (3 μl/min). These perfusion chambers were placed in a culture incubator at 37 °C in 5% CO2. After recording the baseline over 24 h, the perfusion medium was changed to DMEM containing 10 ng/ml recombinant porcine TGF-β2 (R&D Systems) with or without 5 μM SAHA (Sigma-Aldrich) and perfused for 72 h. The IOP was recorded on a computer at a rate of 1 Hz. The aqueous humor outflow facility (μl/min/mmHg) was calculated from the perfusion rate (3 μl/min) and the IOP (mmHg).

### Animal experiments

All experiments were conducted according to the Association for Research in Vision and Ophthalmology Statement for the Use of Animals in Ophthalmic and Vision Research and were approved by the Animal Care and Use Committee at Kumamoto University. Ten male Japanese white rabbits (KBT Oriental, 12–16 weeks old) were used for the animal experiments. All animals were housed under a 12-h dark/light cycle (light from 07:00 to 19:00) at 22 °C ± 1 deg. C with *ad libitum* food and water. We conducted this animal study as described previously (60). Drug administration and IOP measurement were blinded. The anterior chamber volume of the rabbit was estimated to be 300 μl. The final concentrations of the drugs in the anterior chamber were 20 ng/ml TGF-β2 and 5 μM SAHA. The rabbits received intracameral injections of 30 μl TGF-β2 (200 ng/ml) or a mixture of TGF-β2 (200 ng/ml) and SAHA (50 μM) in one eye under sedation (20 μg/kg medetomidine, i.m., Kyoritsu Seiyaku Corporation). The other eye was administered intracamerally with same amount of phosphate-buffered saline (PBS) and used as a control. Five rabbits were tested in each group. Intracameral injection was performed with a microsyringe with a 30 G needle. After drug administration, sedation was reversed using atipamezole (100 μg/kg, i.m., Kyoritsu Seiyaku Corporation). IOP was measured before treatment and at 1, 3, 6, 24, and 48 h after treatment using TONOVET (Icare) in conscious rabbits. The IOP showed the average value of three measurements at each time point, and the difference between the administered eye and the contralateral eye was calculated (ΔIOP).

### Cell culture

Primary MTM and Schlemm’s canal endothelial (MSC) cells were isolated from eyes as previously described (61). Enucleated eyes of cynomolgus monkeys were obtained from Shin Nippon Biomedical Laboratories. MTM and MSC cells were cultured in low-glucose DMEM (FUJIFILM Wako Pure Chemical) in the presence of 10% FBS, glutamine (2 mM), penicillin (100 U/ml), streptomycin (100 μg/ml), and amphotericin B (0.5 μg/ml) at 37 °C in 5% CO2. Cells were used after 3 to 5 passages. HTM cells were purchased from ScienCell Research Laboratories. HTM cells were cultured in low-glucose DMEM (FUJIFILM Wako Pure Chemical) in the presence of 10% FBS, glutamine (2 mM), penicillin (100 U/ml), and streptomycin (100 μg/ml) at 37 °C in 5% CO2. Cells were used after 3 to 6 passages. The identity of TM cells was confirmed by dexamethasone-induced myocilin expression *via* western blotting or real-time RT-PCR.

### Measurement of monolayer transendothelial electrical resistance (TEER)

TEER was measured using the Millicell-ERS (Merck Millipore) as previously described (58). MTM and MSC cells were cultured to confluence on a Transwell polyester membrane insert (0.4-μm pore size, 6.5-mm diameter; Corning Inc) on 24-well culture plates and serum starved overnight. MTM and MSC cells were treated with human recombinant TGF-β2 with or without SAHA. TEER was measured at 24, 48, and 72 h after treatment. Each experiment was performed at least three times.

### Cell viability assay

We examined cell viability using the WST-8 assay (Cell Counting Kit-8, CCK-8; Dojindo), as previously described (62). MTM and MSC cells were seeded on 96-well plates at a density of 1 × 104 cells per well and incubated for 24 h. After serum starvation for 24 h, TGF-β2 with or without SAHA was added to the cells. At 72 h after the addition of the reagents, the TM cells were incubated with 10 μl of CCK-8 solution for 2 h. The absorbance of WST-8 formazan at 450 nm was measured according to the number of live cells using a microplate reader (Multiskan FC, Thermo Fisher Scientific). Cell viability is presented as the relative change compared with the control.

### Cytotoxicity assay

We examined cytotoxicity using a Cytotoxicity LDH Assay Kit-WST (Dojindo) as previously described (62). We evaluated the cytotoxicity under the same conditions as the cell viability assay. The cells were added to the working solution (100 μl/well) and incubated for 30 min at room temperature under shaded conditions. The cells were then added to the stop solution, and the absorbance at 492 nm was measured as lactate dehydrogenase (LDH) activity using a microplate reader. Cytotoxicity was measured as the relative change with respect to the positive control (lysis-buffer-treated cells).

### Western blotting

Western blotting was performed as described previously (59). After TGF-β2 and SAHA treatment, cell lysates were prepared from the MTM, MSC, and HTM cells. Loading samples were prepared from the cell lysates with NuPAGE LDS sample buffer and dithiothreitol (Thermo Fisher Scientific). Samples were loaded onto a polyacrylamide gel, and proteins were separated by sodium dodecyl sulfate–polyacrylamide gel electrophoresis. These proteins were transferred onto polyvinylidene difluoride membranes by electroblotting. The membranes were blocked with 2% bovine serum albumin (BSA; FUJIFILM Wako Pure Chemical), or 5% skim milk (Nacalai Tesque) in Tris-buffered saline containing 0.1% Tween-20 (TBS-T) for 1 h at room temperature, and then incubated with primary antibodies (see Table S1) diluted with 5% BSA or 5% skim milk in TBS-T overnight at 4 °C. After washing with TBS-T, the membranes were incubated with horseradish peroxidase (HRP)-conjugated secondary antibodies for 30 min at room temperature. The chemiluminescence signal was detected using ECL Prime or ECL Select western blotting detection reagent (GE Healthcare) and a luminescence imager (LAS 4000 mini; FUJIFILM). All membranes were stripped of antibodies using WB stripping solution (Nacalai Tesque) and then incubated with anti-β-actin antibody followed by HRP-conjugated rabbit IgG antibody as a loading control. The densitometry of the immunoreactive bands was analyzed using Image J software (National Institutes of Health).

### Real time RT-PCR

RNA samples were prepared from cells using the NucleoSpin RNA Kit (Takara Bio), according to the manufacturer’s instructions. The concentration of RNA samples was ascertained using a DS-11 NanoPad spectrophotometer (DeNovix). Reverse transcription and quantitative PCR were performed as previously described (63). Relative expression of the target mRNAs was compared with the control samples using the comparative threshold cycle method; glyceraldehyde-3-phosphate dehydrogenase was used as an endogenous control. The primer sequences used are listed in Tables 1 and 2.

**Table 1:** Primer sequences for human genes examined by quantitative RT-PCR

Gene	Primer sequences (5′ to 3′)	Product size (bp)
*FN1*	F: CGGTGGCTGTCAGTCAAAG R: AAACCTCGGCTTCCTCCATAA	130
*COL1A1*	F: GTGCGATGACGTGATCTGTGA R: CGGTGGTTTCTTGGTCGGT	119
*ACTA2*	F: GTGTTGCCCCTGAAGAGCAT R: GCTGGGACATTGAAAGTCTCA	109
*PTEN*	F: AGGACCAGAGACAAAAAGGGAGT R: TCATCTTGTGAAACAACAGTGCCA	121
*BMP7*	F: GGAACGCTTCGACAATGAGAC R: GCAGGAAGAGATCCGATTCCC	86
*GAPDH*	F: GCACCGTCAAGGCTGAGAAC R: TGGTGAAGACGCCAGTGGA	138

Abbreviations: ACTA2, actin alpha 2 (smooth muscle); BMP7, bone morphogenic protein 7; COL1A1, collagen type I alpha 1 chain; FN1, fibronectin 1; GAPDH, glyceraldehyde 3-phosphate dehydrogenase; PTEN, phosphatase and tensin homolog.

**Table 2:** Primer sequences for monkey genes examined by quantitative RT-PCR

Gene	Primer sequences (5′ to 3′)	Product size (bp)
*FN1*	F: ACAAGCGTGTCTCTCTGCC R: CCAGGGTGATGCTTGGAGAA	149
*COL1A1*	F: CAAGGTGTTGTGCGATGACG R: CTCCCTTGGGTCCCTCGAC	149
*COL4A1*	F: TTTGGCTCGCCACCATAGAG R: TCATACAGACTTGGCAGCGG	108
*ACTA2*^a^	F: GTGTTGCCCCTGAAGAGCAT R: GCTGGGACATTGAAAGTCTCA	109
*PTEN*	F: TGGCGGAACTTGCAATCCTCA R: TTGTCTTCCCGTCGTGTGGG	90
*PDPK1*	F: GGCTCGAGAACTGGCAACCT R: GCGACATGACATCCCGCTCT	113
*PIK3CA*	F: AATCCCAGGTGGAATGAATGGC R: GCACCCTTTCGGCCTTTAACA	107
*PHLPP1*	F: CGACAAGTCTCCAAGGTTGC R: AAGAGGTTGGCAGGCAGATG	86
*BMP7*	F: AGCTTCGTCAACCTCGTGGAA R: TTGTCGAAGCGTTCCCGGAT	158
*GAPDH*	F: TCGTCATCAATGGAAGCCCC R: AAATGAGCCCCAGCCTTCTC	136

Abbreviations: ACTA2, actin alpha 2 (smooth muscle); BMP7, bone morphogenic protein 7; COL1A1, collagen type I alpha 1 chain; COL4A1, collagen type IV alpha 1 chain; FN1, fibronectin 1; GAPDH, glyceraldehyde 3-phosphate dehydrogenase; PDPK1, 3-phosphoinositide-dependent protein kinase 1; PHLPP1, PH domain and leucine-rich repeat protein phosphatase 1; PIK3CA, phosphatidylinositol-4,5-bisphosphate 3-kinase catalytic subunit alpha; PTEN, phosphatase and tensin homolog.

a Monkey *ACTA2* uses the same primer as human.

### Immunocytochemistry

Immunocytochemical analyses were conducted as described previously (58). The cells were fixed with 4% (v/v) paraformaldehyde in PBS for 15 min at room temperature and then washed with cytoskeletal buffer (10 mM 2-morpholinoethanesulfonic acid potassium salt, 150 mM NaCl, 5 mM EGTA, 5 mM MgCl2, and 5 mM glucose, pH 6.1). For permeabilization, the cells were treated with 0.5% (v/v) Triton X-100 in PBS for 12 min at room temperature. The cells were then blocked with serum buffer (10% FBS and 0.2 mg/ml sodium azide in PBS) at 4 °C for at least 2 h before being treated with primary antibodies (see Table S2) at 4 °C overnight. Afterward, the cells were incubated with anti-mouse or anti-rabbit IgG secondary antibody labeled with Alexa Fluor 488 or 546 phalloidin at room temperature for 30 min. After mounting with VECTASHIELD mounting medium containing 4′, 6-diamidino-2-phenylindole (DAPI; Vector Laboratories), the cells were observed under an all-in-one epifluorescence microscope (BZ-X710; Keyence). We performed immunostaining in at least three independent experiments.

### RNA interference targeting PTEN

siRNA transfection was conducted as described previously. The PTEN siRNA was the Silencer Select Pre-designed siRNA (Ambion, Thermo Fisher Scientific): sense, 5′-GCAUACGAUUUUAAGCGGAtt-3′; antisense, 5′-UCCGCUUAAAAUCGUAUGCag-3′. The cells were cultured to 50 to 70% confluence before transfection. The control or PTEN siRNA was transfected into cells using Lipofectamine RNAiMAX (Thermo Fisher Scientific) according to the manufacturer’s protocol. The final concentration of siRNA in the culture medium was 10 nM. At 48 h after siRNA transfection, the cells were treated with TGF-β2 and SAHA for western blotting and real-time PCR.

### Statistical analysis

The results are expressed as means ± standard deviation (SD). All data were analyzed using JMP statistical software (version 14.3.0; SAS Institute). A statistical comparison of multiple groups was conducted using the Tukey–Kramer honest significant difference (HSD) test. In all analyses, differences were considered statistically significant at *p* < 0.05.

## Data availability

All data described are contained within this article and the supporting information.

## Supporting information

This article contains supporting information.

## Conflict of interest

T. F., None; M. I.-M., None; S. I., None; H. T., Alcon Japan (F), Kowa (F, C), Merck Sharp & Dohme Corp (C), Otsuka Pharmaceutical (F), Pfizer Japan (F), Santen Pharmaceutical (F), Senju Pharmaceutical (F); T. I., Alcon Japan (F), Kowa (F), Novartis Pharmaceutical (F), Otsuka Pharmaceutical (F), Pfizer Japan (F), Santen Pharmaceutical (F), Senju Pharmaceutical (F).
