# Supplementary material for: Suberoylanilide hydroxamic acid (SAHA) inhibits transforming growth factor-beta 2-induced increases in aqueous humor outflow resistance
Source: J Biol Chem. 2021 Aug 11;297(3):101070. doi: 10.1016/j.jbc.2021.101070 (PMC8406002; doi:10.1016/j.jbc.2021.101070)
Supplement: Supplemental Figures S1–S3 and Tables S1, S2 [file mmc1.docx]

**Supporting Table S1. Antibodies for Western blot analysis**

| Target protein | Catalog Number | Dilution | Source |
| --- | --- | --- | --- |
| Fibronectin | ab6328 | 1/2000 | Abcam, Cambridge, UK |
| Collagen type I | ab138492 | 1/2000 |  |
| Collagen type IV | ab6586 | 1/1000 |  |
| p-Smad3(S423/425) | ab52903 | 1/2000 |  |
| α-SMA | 19245 | 1/2000 | Cell Signaling Technology, Danvers, MA |
| N-cadherin | 4061 | 1/1000 |  |
| Snail | 3879 | 1/1000 |  |
| Smad2/3 | 8685 | 1/1000 |  |
| p-Smad2 (S465/467) | 3108 | 1/1000 |  |
| ERK1/2 | 9102 | 1/1000 |  |
| p-ERK1/2 (T202/Y204) | 9101 | 1/1000 |  |
| Akt | 9272 | 1/1000 |  |
| p-Akt (S473) | 9271 | 1/1000 |  |
| PTEN | 9559 | 1/1000 |  |
| Histone H3 | 4499 | 1/2000 |  |
| Ac-H3 (K9) | 9649 | 1/1000 |  |
| Ac-H3 (K14) | 7627 | 1/1000 |  |
| Ac-H3 (K18) | 13998 | 1/1000 |  |
| Ac-H3 (K27) | 8173) | 1/1000 |  |
| Ac-H3 (K56) | 4243 | 1/1000 |  |
| Ac-H4 (K5) | 8647 | 1/1000 |  |
| Ac-H4 (K8) | 2594 | 1/1000 |  |
| Ac-H4 (K12) | 13944 | 1/1000 |  |
| Anti-rabbit IgG HRP | 7074 | 1/2000 |  |
| Anti-mouse-IgG HRP | 7076 | 1/2000-5000 |  |
| Histone H4 | 07-108 | 1/1000 | Sigma-Aldrich, Merck KGaA, Darmstadt, Germany |
| β-catenin | C2206 | 1/4000 |  |
| β-actin | A5441 | 1/10000 |  |

**Supporting Table S2. Antibodies for immunocytochemistry.**

| Target protein | Catalog Number | Dilution | Source |
| --- | --- | --- | --- |
| Collagen type I | ab138492 | 1/2000 | Abcam, Cambridge, UK |
| Collagen type IV | ab6586 | 1/1000 |  |
| α-SMA | 19245 | 1/200 | Cell Signaling Technology, Danvers, MA |
| Smad2/3 | 8685 | 1/800 |  |
| β-catenin | C2206 | 1/1000 | Sigma-Aldrich, Merck KGaA, Dermstadt, Germany |
| ZO-1 | 617300 | 1/100 | Invotrogen, Thermo Fisher Scientific, Rockford, IL, USA |
| Alexa Fluor™ 488 goat anti-mouse IgG (H+L) | A11001 | 1/1000 |  |
| Alexa Fluor™ 546 phalloidin | A22283 | 1/200 |  |


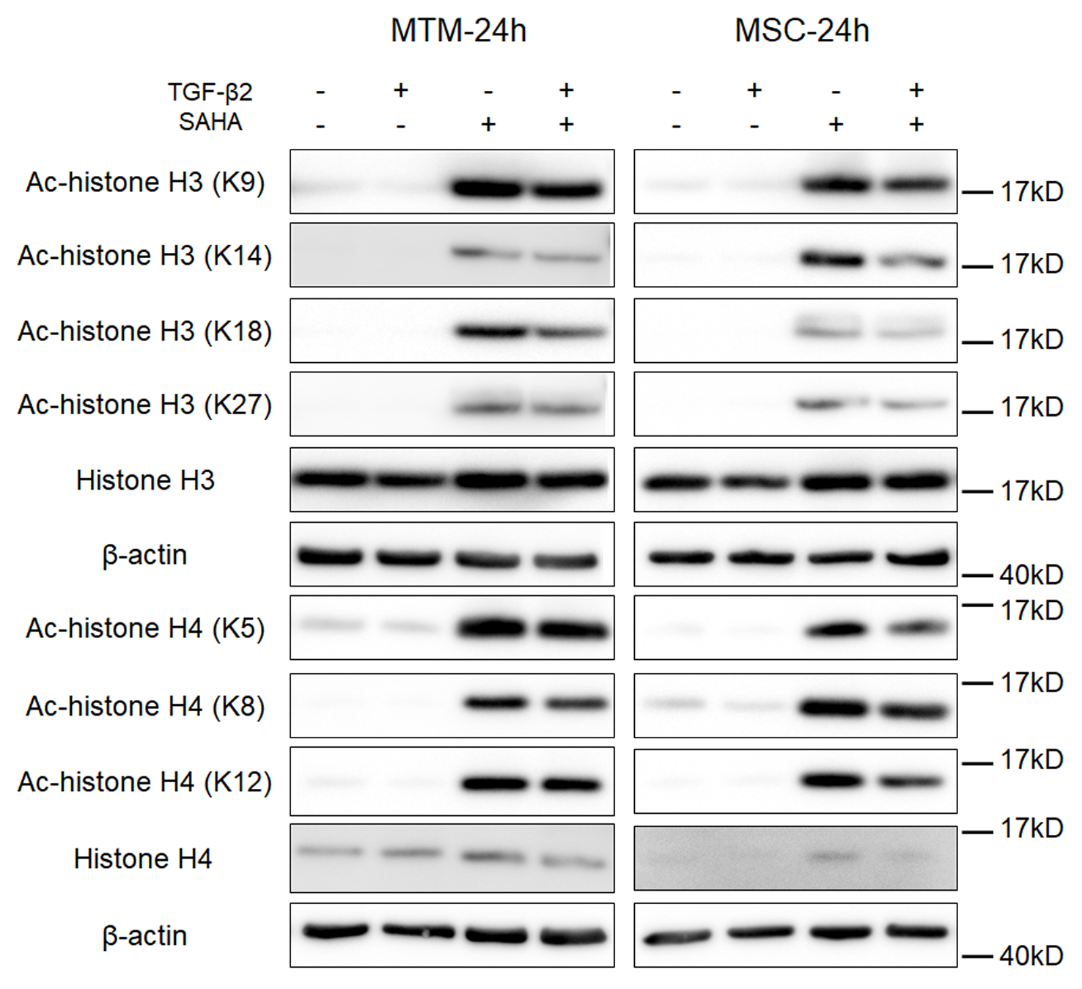


**Supporting Figure S1. The effects of TGF-β2 and SAHA on histone acetylation in MTM and MSC cells.** MTM and MSC cells were treated with 5 ng/mL TGF-β2 and/or 5 µM SAHA for 24 h. Acetyl histones (Ac-histone) H3 and H4 were detected by Western blotting. Data show the representative bands of Ac-histone and total histone.


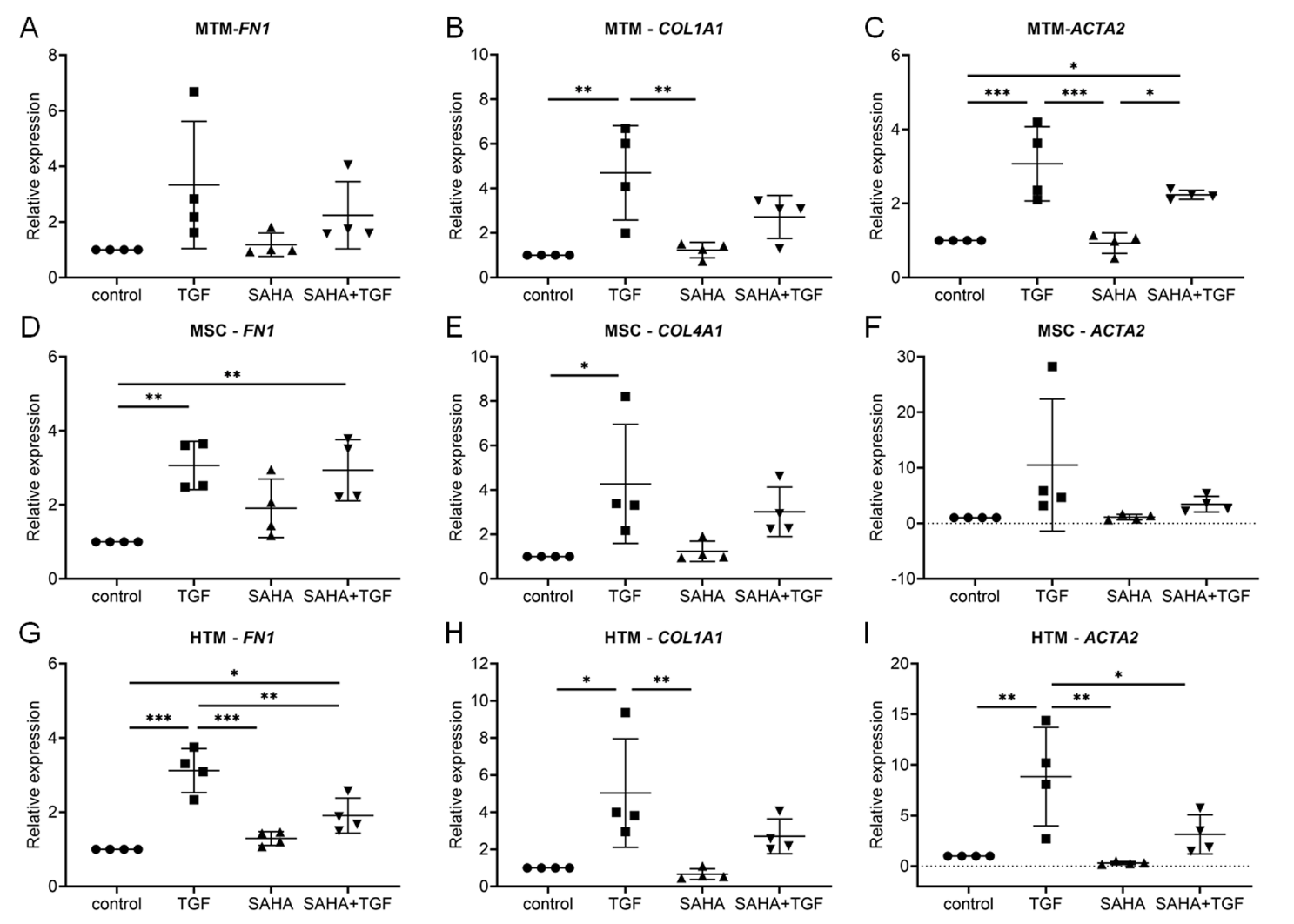


**Supporting Figure S2. The effects of TGF-β2 and SAHA on mRNA expression in MTM, MSC, and HTM cells.** MTM (A–C), MSC (D–F), and HTM (G–I) cells were treated with 5 ng/mL TGF-β2 and 5 µM SAHA for 24 h. The mRNA expression levels of *FN1* (fibronectin 1; A, D, and G), *COL1A1* (collagen type I alpha 1 chain; B and H), *ACTA2* (α-SMA; C, F, and I), and *COL4A1* (collagen type IV alpha 1 chain; E) were evaluated by real-time RT-PCR. Data are presented as means ± SD (n = 4). * *p* < 0.05, ** *p* < 0.01, and *** *p* < 0.001, Tukey–Kramer HSD.


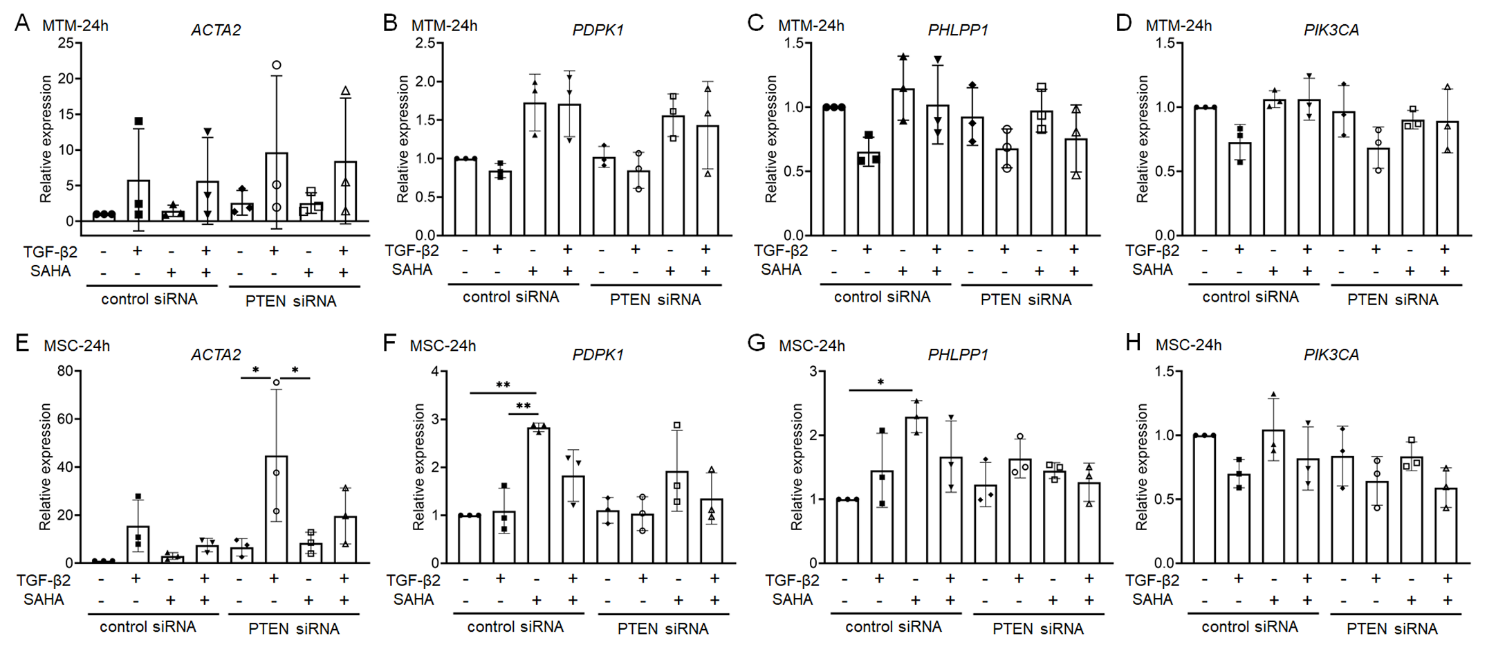


**Supporting figure S3. The effects of PTEN knockdown on mRNA expression after TGF-β2 and SAHA treatment of MTM and MSC cells.** MTM (A–D) and MSC (E–H) cells were treated with 5 ng/mL TGF-β2 and 5 µM SAHA for 24 h. The mRNA expression levels of *ACTA2* (A, E), *PDPK1* B, I), *PHLPP1* (C, J), and *PIK3CA* (D, K) were evaluated by real-time PCR. Data are presented as means ± SD (n = 3). * *p* < 0.05, and ** *p* < 0.01, Tukey–Kramer HSD. ACTA2, α-SMA; PDPK1, 3-phosphoinositide dependent protein kinase 1; PHLPP1, PH domain and leucine rich repeat protein phosphatase 1; PIK3CA, phosphatidylinositol-4,5-bisphosphate 3-kinase catalytic subunit alpha.
